# BAHD acyltransferase from dragon fruit enables production of phyllocactin in engineered yeast

**DOI:** 10.1093/femsyr/foae041

**Published:** 2025-02-10

**Authors:** Christiane Glitz, Jane Dannow Dyekjær, Sophia Mattitsch, Mahsa Babaei, Irina Borodina

**Affiliations:** The Novo Nordisk Foundation Center for Biosustainability, Technical University of Denmark, Kemitorvet Building 220, DK-2800 Kgs. Lyngby, Denmark; The Novo Nordisk Foundation Center for Biosustainability, Technical University of Denmark, Kemitorvet Building 220, DK-2800 Kgs. Lyngby, Denmark; The Novo Nordisk Foundation Center for Biosustainability, Technical University of Denmark, Kemitorvet Building 220, DK-2800 Kgs. Lyngby, Denmark; The Novo Nordisk Foundation Center for Biosustainability, Technical University of Denmark, Kemitorvet Building 220, DK-2800 Kgs. Lyngby, Denmark; The Novo Nordisk Foundation Center for Biosustainability, Technical University of Denmark, Kemitorvet Building 220, DK-2800 Kgs. Lyngby, Denmark

**Keywords:** *Saccharomyces cerevisiae*, *Yarrowia lipolytica*, *in vivo* enzyme screening, betalains, phyllocactin, natural food colours

## Abstract

Microbial fermentation can provide a sustainable and cost-effective alternative to traditional plant extraction to produce natural food colours. Betalains are a class of yellow to red water-soluble pigments. Even though over 80 betalain variants are known, betanin is the only betalain available as a food colourant on the market. Many variants are acylated, which can enhance their stability and change the hue, but very few acyltransferases responsible for the acylation are known. Therefore, we mined the transcriptomes of *Celosia argentea* var. *cristata* and *Hylocereus polyrhizus* for BAHD acyltransferases, enzymes likely involved in betalain acylation. *In vivo* screening of the enzymes in betanin-producing *Saccharomyces cerevisiae* revealed that the acyltransferase HpBAHD3 from *H. polyrhizus* malonylates betanin, forming phyllocactin (6′-O-malonyl-betanin). This is the first identification of a BAHD acyltransferase involved in betalain biosynthesis. Expression of HpBAHD3 in a *Yarrowia lipolytica* strain engineered for high betanin production led to near-complete conversion of betanin to phyllocactin. In fed-batch fermentation, the strain produced 1.95 ± 0.024 g/l phyllocactin in 60 h. This study expands the range of natural food colourants produced through microbial fermentation and contributes to elucidating the biosynthesis pathway of acylated betalains.

## Teaser

Discovery of an acyltranferase from red dragon fruit that enables recombinant production of the red pigment phyllocactin in *Saccharomyces cerevisiae* and *Yarrowia lipolytica*.

## Introduction

Natural colours are derived from various natural sources, such as plants, insects, or microorganisms. In the food industry, colourants play a crucial role in the appeal and perception of food and beverages, and natural or synthetic dyes are commonly used to achieve the desired colour and make the products more attractive and appetizing (Spence 2015). Nature offers a great variety of pigments that can be used for food applications, spanning the whole colour spectrum. Many of these pigments are also linked to health benefits, including antioxidant properties, cardiovascular protection, and anti-inflammatory and anti-diabetic effects (Sigurdson et al. 2017, de Mejia et al. 2020). With increasing concern towards synthetic dyes regarding their safety and sustainability, the demand for these natural food colourants has consistently been rising over the last years (Martins et al. 2016, Oplatowska-Stachowiak and Elliott 2017, Kwon et al. 2022). However, despite their advantages, natural pigments have several limitations compared to synthetic alternatives. Many natural colourants are sensitive to environmental factors such as heat, light, pH, enzymes, and metal ions, leading to reduced stability and a shorter shelf life of the food products (Galaffu et al. 2015). Additionally, the seasonal variability, inconsistent supply, presence of plant-derived off-flavours, and the large amount of raw material needed for pigment extraction negatively affect the sustainability and cost-effectiveness of natural colours, making them more expensive than synthetic options (Delgado-Vargas et al. 2010; European Food Safety Authority (EFSA) 2015; Gebhardt et al. 2020). The heterologous production of natural colours offers a potential solution, combining the benefits of natural colours with a more sustainable and continuous process that can lower production costs. For instance, life-cycle analysis and techno-economic assessment showed that heterologous betanin production with yeast was superior to the traditional extraction process regarding sustainability and economic aspects. Prerequisites, however, are high titres and yields of the biotechnological process (Thomsen et al. 2023). Recent advances in genetic engineering of microorganisms for microbial fermentation of natural (food) colourants have been summarized by Prabowo et al. (2022) and Thomsen et al. (2024).

**Figure 1.**
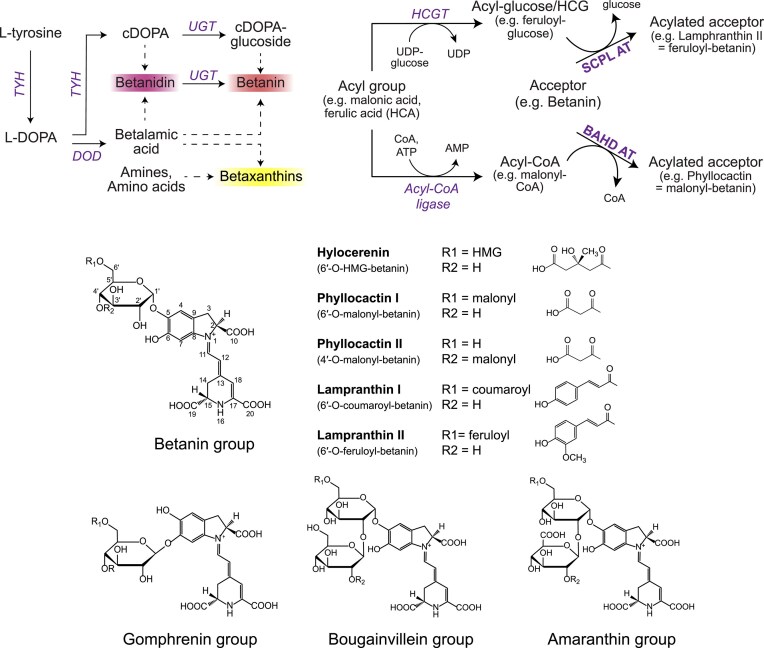
Betalain biosynthesis in plants. (A) Simplified biosynthesis pathway of betaxanthins and betanin in plants. Three enzymes are involved in betanin biosynthesis: a tyrosine hydroxylase (TYH), a DOPA-4,5-extradiol dioxygenase (DOD), and a UDP-glycosyltransferase (UGT) that is either active on cDOPA or on betanidin. Dashed arrows represent spontaneous reactions. (B) Classification of betalain variants in four groups: betanin, gomphrenin, bougainvillein, and amaranthin groups. Examples of acylated variants of the betanin group are shown. (C) Two potential mechanisms for betacyanin acylation have been proposed: acylation by serine-carboxy-peptidase-like (SCPL) acyltransferases (1) or acylation by BAHD acyltransferases (2). HCA = hydroxycinnamic acid; HCGT = hydroxycinnamate glucosyltransferase; HCG = hydroxycinnamoyl glucose; BAHD AT = BAHD acyltransferase; SCPL AT = serine-carboxy-peptidase-like acyltransferase; CoA = coenzyme A.

Alongside anthocyanins, carotenoids, and chlorophylls, betalains are a class of natural pigments present in plants (Tanaka et al. 2008, Kumorkiewicz-Jamro et al. 2021). They cover the yellow-red colour spectrum and derive from L-tyrosine (Fig. 1A). In the first step of the biosynthesis pathway, tyrosine is hydroxylated to L-DOPA (3,4-dihydroxy-l-phenylalanine) by a bifunctional tyrosine hydroxylase (TYH) (Steiner et al. 1999, Deloache et al. 2015, Babaei et al. 2023). In the second step, a DOPA-4,5-extradiol dioxygenase (DOD) converts L-DOPA into 4,5-cyclo-DOPA, which undergoes spontaneous cyclisation to betalamic acid, the common chromophore of betalains (Christinet et al. 2004, Takahashi et al. 2009). In parallel, L-DOPA is oxidized to L-dopaquinone by the TYH and then spontaneously cyclizes to cyclo-DOPA (cDOPA). Betalamic acid can spontaneously condense with cDOPA, forming the unstable violet-red intermediate betanidin, the base structure of betacyanins (Steglich and Strack 1990). In the last enzymatic step, betanidin is glycosylated at the C5-hydroxyl position by a regioselective UDP-glycosyltransferase (UGT), forming betanin (Heuer and Strack 1992). Alternatively, cDOPA can first be glycosylated by a UGT to cDOPA-5-O-glucoside, which then condenses with betalamic acid to betanin (Sasaki et al. 2005). Betalamic acid can also condense with primary and secondary amines to form yellow betaxanthins.

The red pigment betanin is the best-known compound of the betalain family. Betanin is the primary pigment in beetroot and the only betalain on the market. Under the name ‘Beetroot Red’ (E162), it is commonly used in confectionery, beverages, meat replacement, or dairy products (European Food Safety Authority (EFSA) 2015). Betalains are not limited to beetroot but are found in various plants within the Caryophyllales order, which includes food crops such as spinach, quinoa, beetroot, or amaranth; ornamental plants such as cockscomb, bougainvillea, globe amaranth, and 4 o'clock flower; and cacti such as prickly pear and dragon fruit (Steglich and Strack 1990, Carreón-Hidalgo et al. 2022). While betanin is the most used betalain, over 80 more variants of this pigment family have unambiguously been identified, of which around 50 are classified as betacyanins (Coy-Barrera 2020, Kumorkiewicz-Jamro et al. 2021). The derivatisation of the 5-O or 6-O position of cDOPA through acylation or glycosylation enables this wide variety of betacyanin structures. Betacyanin variants are categorized into four groups: betanin-, gomphrenin-, bougainvillein-, and amaranthin-type pigments (Fig. 1B) (Steglich and Strack 1990, Polturak and Aharoni 2018). Despite being known for many years, no betalain other than betanin is used as a food colourant on an industrial scale. Several review articles provide a detailed overview of the most relevant betalain variants, including those by Steglich and Strack (1990), Kumorkiewicz-Jamro et al. (2021), and Sadowska-Bartosz and Bartosz (2021).

The physiochemical properties of betalains, such as hue, stability, or antioxidant activity, are influenced by the derivatisation pattern of the betanidin backbone. Glycosylation of betanidin, resulting in betanin (betanidin-5-O-β-glucoside) or gomphrenin (betanidin-6-O-β-glucoside), leads to a hypsochromatic shift (lower λ_max_). Further glycosylation has not been found to influence the max. absorbance and has little effect on the compound's stability (Huang and von Elbe 1986, Schliemann and Strack 1998). Esterification with aliphatic acyl moieties also has little impact on the absorbance maximum but can enhance pigment stability, while acylation with aromatic acids leads to a bathochromatic shift (higher λ_max_) and a more pronounced stability increase (Schliemann and Strack 1998, Herbach et al. 2006a). Intramolecular stacking and the protection of the aldimine bond by the acyl group are considered responsible for this enhanced stability.

Acylation is a widespread modification of plant secondary metabolites that expands metabolite diversity (Mugford and Milkowski 2012). Acylation describes the transfer of an acyl group via an activated donor molecule to an acceptor molecule, forming an acyl conjugate. These reactions are catalysed by acyltransferases (ATs) (EC 2.3.1.). The acylation pathway for betalains has not been fully elucidated but two potential acylation mechanisms have been proposed: acylation by serine-carboxy-peptidase-like (SCPL) ATs or by BAHD ATs. SCPL ATs utilize glucose esters, such as hydroxycinnamoyl glucose (HCG), as activated acyl donors (Fig. 1C (1)). These are synthesized by glycosylation of hydroxycinnamic acids (HCA) by hydroxycinnamate glucosyltransferases (HCGT) (Steffens 2000, Mugford and Milkowski 2012). Bokern et al. (1992) identified an AT in *Chenopodium rubrum* and in *Lampranthus sociorum* that catalysed the transfer of coumaric acid and ferulic acid from their respective hydroxycinnamoyl-glucose ester to amaranthin, yielding celosianin I and II. Later, HCGTs were found in *Gomphrena globosa* and *Mirabilis jalapa*, indicating that SCPLs are involved in betalain acylation in these plants (Matsuba et al. 2008, Polturak et al. 2018). Alternatively, betalains may undergo acylation by acyl-CoA-dependent ATs from the BAHD superfamily (Fig. 1C (2)). BAHD ATs are involved in a variety of acylation reactions of natural products, including anthocyanins, but their role in betalain acylation has yet to be demonstrated (D’Auria 2006, D’Auria et al. 2007, Luo et al. 2022). Unlike SCPLs, which are primarily associated with aromatic acids, BAHD AT can transfer both aromatic and aliphatic acids (Bontpart et al. 2015).

Heterologous production of betanin has been successfully engineered in several plants, including *Nicotiana benthamiana, Solanum lycopersicum*, and *Oryza sativa japonica* (Polturak et al. 2016, Tian et al. 2020, Grützner et al. 2021), in *Saccharomyces cerevisiae* (Grewal et al. 2018, Babaei et al. 2023, Zhang et al. 2023, Li et al. 2024), and *E. coli* (Hou et al. 2020). A recent study reported the production of 1.91 g/l betanin in filamentous fungi, exceeding the previous highest titre of 1.3 g/l achieved with *Yarrowia lipolytica* (Thomsen et al. 2023, Tong et al. 2024). However, all these previous efforts focused on betanin. With this study, we thus aimed to build on the existing research in recombinant betanin production by extending the betalain portfolio with acylated betacyanins.

## Materials and methods

### Assembly and annotation of *Hylocereus polyrhizus* and *Celosia argentea* var. *cristata* transcriptomes

We downloaded paired-end Illumina HiSeq 2500 transcriptomic RNA-seq raw reads of red pulp (42nd day) and white pulp (28th day) samples from *H. polyrhizus* from NCBI (SRR3203780) and (SRR2924904), respectively. FASTQ files were generated using NCBI's SRAToolKits.2.10.4 FASTQ-dump tool. We used FastQC (Andrews 2010) to do quality control and check the reads for overrepresented sequences. The reads were trimmed with TrimGalore-0.6.5 using a phred33 quality score (Krueger 2019). The trimmed paired-end FASTQ reads for each of the red and white pulp were *de novo* assembled using Trinity v2.9.1 (Grabherr et al. 2011). Calculating the assembly statistics with the TrinityStats.pl script in Trinity v2.9.1 resulted in a median transcript contig length of 331 with an N50 value of 474. In addition, the trimmed, paired-end FASTQ file for the red and white pulp was concatenated and then *de novo* assembled using Trinity v2.9.1. Next, we calculated the expression levels of red and white reads with respect to the combined assembled transcriptome. We used the align_and_estimate_abundance.pl script in Trinity v2.9.1 using Bowtie as the alignment method and RSEM as the abundance estimation method. A counts matrix for the isoforms was generated using the abundance_estimates_to_matrix.pln script and the differential expression analysis was carried out using the run_DE_analysis.pl script using 0.1 as dispersion, as there are no biological replicates. The obtained FPKM values are normalized using the run_TMM_normalization_write_FPKM_matrix.pl script. To identify the most differentially expressed transcript, the script analyse_diff_expr.pl was used. Finally, the longest open reading frames (ORFs) were extracted using TransDecoder (Haas 2023). Then, we annotated the transcriptome by blasting the transcripts and ORFs into local Uniprot databases. In addition, we extracted the PFAM domains for the ORFs. The results were loaded into an SQLite database using Trinotate v3.2.0 (Haas 2019). Finally, we merged the expression levels into an annotation table. For *Celosia argentea*, we downloaded paired-end Illumina HiSeq 2500 transcriptomic RNA-seq raw reads from a plant sample of *C. argentea* var. *cristata* (NCBI: SRR9095475) and proceeded as done with the individual *H. polyrhizus* reads.

### Query for BAHD AT enzymes from plants

To find novel BAHD ATs for production of acylated betalains in yeast, we BLASTed the protein sequences of the malonyl-CoA:isoflavone 7-O-glucoside-6″-O-malonyltransferase from *Glycine max* (soybean, UniProtID: A7BIC9_SOYBN) and the malonyl-CoA:anthocyanidin 5-O-glucoside-6″-O-malonyltransferase from *Arabidopsis thaliana* (mouse-ear cress, UniProtID: 5MAT_ARATH) into the assembled *C. argentea* flower transcriptome (NCBI: SRR9095475) and the differentially expressed assembled transcriptomes of *H. polyrhizus* (red pulp stage dragon fruit sample, NCBI: SRR3203780) and *H. polyrhizus* (white pulp stage dragon fruit sample, NCBI: SRR2924904) (D’Auria et al. 2007). In addition, sequences belonging to PFAM PF02458 and annotated as BAHD were extracted from the *C. argentea* transcriptome and the differentially expressed *H. polyrhizus* transcriptome. The resulting sequence hits were filtered for presence of conserved HXXXD motif, and those starting with a start codon were extracted and identical sequences removed. Of the remaining sequences from *C. argentea*, the three highest expressed genes were selected and named CcBAHD1-CcBAHD3. Of the remaining sequences from *H. polyrhizus*, six were selected based on their expression level in red dragon fruit or because of significantly (2-fold) higher expression in red pulp stage dragon fruit than in white pulp stage dragon fruit and named HpBAHD1–HpBAHD6. These nine genes were ordered codon-optimized for *S. cerevisiae* as gene strings (Table 1). Heterologous genes were synthesized as synthetic gene strings and codon-optimized for *S. cerevisiae* or *Y. lipolytica* by Twist Bioscience (USA). The corresponding amino acid and nucleotide sequences can be found in Supplementary File S2. Oligonucleotides were obtained from Integrated DNA Technologies (Supplementary File S2).

**Table 1.** BAHD ATs screened for production of acylated betalain variants in *S. cerevisiae*.

Name	Origin	Strain ID *S. cerevisiae*	Strain ID *Y. lipolytica*
CcBAHD1	*Celosia argentea*	ST13941	
CcBAHD2	*Celosia argentea*	ST13942	
CcBAHD3	*Celosia argentea*	ST13943	
HpBAHD1	*Hylocereus polyrhizus*	ST13944	
HpBAHD2	*Hylocereus polyrhizus*	ST13945	
HpBAHD3	*Hylocereus polyrhizus*	ST13946	ST14103
HpBAHD4	*Hylocereus polyrhizus*	ST13947	
HpBAHD5	*Hylocereus polyrhizus*	ST13948	
HpBAHD6	*Hylocereus polyrhizus*	ST13949	

The candidate genes were found in the transcriptomes of the indicated plant species and selected based on their expression levels. The yeast strains in which the respective BAHD AT was integrated are listed.

### Media and cultivations

*E. coli* DH5α strains, used for cloning and plasmid propagation, were cultivated in lysogeny broth (LB), supplemented with 100 mg/l ampicillin, at 37°C, 200 rpm. During strain construction, *S. cerevisiae* strains were grown in yeast peptone dextrose media (YPD), supplemented with 200 mg/l geneticin (G418) for Cas9-plasmid selection, *Y. lipolytica* strains were grown in YPD without G418. After transformation, yeast strains were plated on synthetic complete (SC) agar plates prepared with 76 mg/l of the standard amino acids, 380 mg/l leucine, 18 mg/l adenine, 7 mg/l inositol, and 8 mg/l *p*-aminobenzoic acid (*p*ABA). For small-scale cultivations in 24-well plates, mineral media (MM), buffered with potassium phosphate (KH_2_PO_4_), was prepared with 7.5 g/l (NH_4_)_2_SO_4_, 14.4 g/l KH_2_PO_4_, 0.5 g/l MgSO_4_ 7·H2O, trace metals and vitamins, and 20 g/l glucose and adjusted to pH 6.0 with NaOH (Jensen et al. 2014). The yeast strains were cultivated at 30°C, 250 rpm. For agar plates, 20 g/l agar was added to the respective media.

### Plasmid and strain construction

*Saccharomyces cerevisiae* strains were constructed according to the EasyClone MarkerFree toolkit, *Y. lipolytica* strains were constructed according to the EasyClone YALI toolkit (Jessop-Fabre et al. 2016, Holkenbrink et al. 2018). Promoters and heterologous genes were amplified by PCR with USER-compatible overhangs using Phusion U Hot Start DNA Polymerase (Thermo Fisher Scientific, USA) and assembled with PCR-linearized integration vectors in *E. coli* DH5α by USER Cloning (Bitinaite et al. 2007). The plasmids were purified from *E. coli* using a NucleoSpin plasmid miniprep kit (Macherey Nagel, Germany), and correct plasmid assembly was verified by Sanger sequencing (Eurofins Genomics, LU). Plasmids were linearized with FastDigest NotI (Thermo Fisher Scientific), resulting in a fragment containing the promoter, gene of interest, terminator, and 500–600 bp upstream and downstream homology regions that navigate the integration site in the host genome. The integration fragment and the gRNA were transformed into Cas9-expressing *S. cerevisiae* or *Y. lipolytica* strains with the LiAc method. (Gietz and Schiestl 2007) Transformants were selected on SC plates containing 100 mg/l nourseothricin (*Y. lipolytica*: 250 mg/l nourseothricin) and 200 mg/l G418 (*S. cerevisiae* strains only). Correct integration of the fragment was verified by colony PCR using RedTaq MasterMix (VWR life science).

The *S. cerevisiae* strains used in this study were derived from the haploid strain CEN.PK113-7D (MATa *URA3 HIS3 LEU2 TRP1 MAL2-8c SUC2*). The *Y. lipolytica* strains generated in this study were derived from a W29/CLIB89 (NRRL Y-63746) strain containing a Cas9 expression cassette in the KU70 locus. Glycerol stocks of the created *E. coli* and yeast strains were made by adding glycerol to overnight cultures [25% (v/v)] and storing them at −70°C. All biobricks, plasmids, and strains created for this work are listed in the Supplementary File S2.

### Small-scale cultivation in 24-well plates

Screening of the selected BAHD AT in *S. cerevisiae* was performed in 24-deep well plates. Precultures, inoculated from single colonies on agar plates, were grown for 24 h in 2 ml MM (+*p*ABA) in 24-well plates for 24 h. Two milliliters of MM (without *p*ABA) were inoculated from the precultures in a ratio 1:200 (v/v), corresponding to an OD_660_ of ca. 0.1, and the strains cultivated for 48 h at 30°C and 250 rpm. Afterwards, the cultures’ supernatant and total cell extract (fermentation broth with cells) were analysed. For the supernatant, 1 ml of cultivation broth was centrifuged at 5000 × *g* for 10 min, the supernatant transferred to 1.5 ml reaction tubes, centrifuged again, and stored at −20°C until further use. For the total extract (intracellular + extracellular), 1 ml of cell culture was transferred into a 2 ml microtube (Sarstedt) containing ca. 0.25 ml of 0.5–0.75 mm glass beads. The cells were then disrupted using a Precellys R 24 homogenizer (Bertin Corp.) in five cycles of 5000 rpm for 30 sec, with a cooling step on ice between each lysis cycle. After disruption, the tubes were centrifuged for 10 min at 10 000 g, the supernatant transferred to 1.5 ml reaction tubes, centrifuged again, and finally stored at −20°C. The produced titres were not normalized to the OD_660_ because despite measuring OD at 660 nm instead of 600 nm, there is still overlap with the maximal absorbance of the betacyanins (540 nm), which causes significant deviations in the OD values. The same cultivation protocol was followed to test the enzymes in *Y. lipolytica*, except that the precultures were grown for 48 h.

### Fed-batch fermentations

The fed-batch fermentation was carried out in single-use 250 ml bioreactors in duplicates (AMBR250, Sartorius AG). The *Y. lipolytica* strain ST14103 was streaked from cryostock on YPD agar plates and incubated for 48 h at 30°C. Two milliliters of MM in a pre-culture tube were inoculated with a single colony and incubated with shaking at 250 rpm for 24 h. Afterwards, a baffled shake flask with 50 ml MM was inoculated to OD_660_ = 0.1 and incubated for another 24 h. The culture was centrifuged (3000 × *g*, 10 min), washed twice with Milli-Q water, and concentrated to 10 ml. With this cell suspension, the bioreactors, filled with 100 ml batch media, were inoculated to a starting OD_660_ of 1. The pH was kept at pH 6 with 1 M NaOH, samples were collected automatically every 6 h and immediately frozen (−14°C). Exponential feeding was initiated once the glucose in the batch media was depleted. Afterwards, the glucose concentration in the media and the total betalain concentration (intra- and extracellular) were quantified by HPLC. Medium compositions, operational fermentation parameters, and the raw online and offline data can be found in the Supplementary File S3.

### Plant material and pigment extraction

Fresh *H. polyrhizus* fruits were purchased from ‘FreshLand’. For extraction of pigments from *H. polyrhizus*, a fresh fruit was peeled, the fruit flesh cut into pieces, and crushed with mortar and pestle. The crushed fruit flesh was mixed with 10 mM ascorbic acid and stirred for 1 h at 4°C on a magnetic stirrer. The solid plant residues were removed by centrifugation (4°C, 11 000 × *g*, 5 min), followed by filtration through a 0.45 µm syringe filter, then a 0.2 µm syringe filter. The extracts were stored at −20°C until analysis by HPLC and LC–MS. For analytical comparison with yeast cultures, the extract was diluted up to 1:10 with MM.

### Spectrophotometric quantification of betacyanins

As reported before, betanin and isobetanin were quantified using a commercially available beetroot extract that contains the pigment diluted with dextrin (TCI, Product Number: B0397) (Babaei et al. 2023, Thomsen et al. 2023). Since no commercial standard was available for phyllocactin, it was quantified with a standard made from *H. polyrhizus*. The identification of the compound was achieved with LCMS. To obtain pure phyllocactin, it was purified from the plant extracts by semi-preparative HPLC. Absorbance at 535 nm was measured in a spectrophotometer, and by employing the Beer–Lambert equation, assuming a molar extinction coefficient of ε = 65.000 M/cm, a calibration curve was made (Supplementary Table S1).

### Analytical methods

To quantify the betalains produced by the yeast cultures, the samples were analysed via high-performance liquid chromatography (HPLC) using a Dionex Ultimate 3000 HPLC system (Thermo Fisher Scientific, USA). The samples were run on a Zorbax Eclipse Plus C18 reverse-phased column (particle size 3.5 µm, pore size 95 Å, 4.6 × 100 mm). The column oven temperature was set to 30°C and the flow rate to 1 ml/min, with 10 µl of sample injection. Solvent A was 0.1% formic acid, and solvent B was 100% acetonitrile. The solvent composition was initially A = 98.0% and B = 2.0% for 2 min. A linear gradient was run until A = 90.0% and B = 10.0% at 5.0 min. A second gradient was run until A = 85.0% and B = 15.0% at 8.0 min. At 8.2 min, the column was flushed by setting A = 2.0% and B = 98.0%. At 9.5 min, the initial conditions (A = 98.0%, B = 2.0%) were reset and remained unchanged until the end of the run (11.5 min). The UV-Vis detector was set to capture data at 390, 480, and 540 nm.

Semi-preparative HPLC was performed to obtain pure fractions of phyllocactin for quantification purposes. A volume of 100 µl of sample were injected into a Zorbax Eclipse Plus C18 reverse-phased column (particle size 3.5 µm, pore size 95 Å, 4.6 × 100 mm, kept at 30°C and run with a flow rate of 0.8 ml/min). Solvent A was water + 0.1% formic acid, and solvent B was 100% acetonitrile. The solvent composition was initially set to A = 98.0% and B = 2.0% and kept steady for 2 min. Hereafter, the solvent composition was adjusted following a linear gradient, until reaching A = 90.0% and B = 10.0% at 12 min. Then, the solvent composition was adjusted following a second linear gradient, until reaching A = 80.0% and B = 20.0% at 17 min. The column was then flushed by setting A = 2.0% and B = 98% at 18 min. These conditions were kept steady until 18.5 min and were then returned to the initial conditions of A = 98.0% and B = 2.0% at 19 min, at which point the solvent composition remained unchanged until the end of the run at 20.5 min. All betalains were detected with a UV-VIS detector at a wavelength of 540 nm. To obtain the desired compound fraction, the respective time span in which that compound elutes was selected. The acetonitrile was evaporated and the fractions up-concentrated with a vacuum evaporator to achieve higher concentrations. Afterwards, the fraction was analysed by LC–MS for validation and then used as HPLC standards and to make a calibration curve.

For glucose quantification, the HPLC system was equipped with an Aminex organic acid column kept at 50°C. Isocratic elution was run with 5 mM H_2_SO_4_ at a flow rate of 0.6 ml/min for 30 min. Glucose was detected with an RI detector, 20 µl were injected.

Untargeted LC–UV–tandem mass spectrometry (MS/MS) was performed to identify the betalain variants in plant extracts and yeast cultivations, using a UHPLC Ultimate 3000 binary system (Thermo Fisher Scientific, USA) coupled to a DAD-(ESI)Fusion Orbitrap Mass Spectrometer (Thermo Fisher Scientific, USA). The chromatographic separation was achieved using a Waters ACQUITY BEH C18 column (10 cm × 2.1 mm, 1.7 µm) equipped with an ACQUITY BEH C18 guard column at 30°C and a flow rate of 0.35 ml/min. The mobile phase was composed of Solvent A (Milli-Q water + 0.1% formic acid) and Solvent B (acetonitrile + 0.1% formic acid) and run with a gradient as follows: B = 0.2% for 3 min, followed by a linear increase up to B = 25% within 20 min and kept steady for 1 min. After that, the concentration of B increased to 100% in 4 min and stayed at 100% for 2 min before returning to initial conditions. Re-equilibration time was 2 min. A volume of 1 ul sample was injected. The DAD settings were the following: data collection rate: 10 Hz, wavelength range: 190-600 nm, bandwidth: 2 nm. MS acquisition was set to positive-heated electrospray ionization (+HESI) mode with a voltage of 3500 V, acquiring in full MS/MS spectra (data-dependent acquisition-driven MS/MS) with a mass range of 70–1000 Da. DAD acquisition settings were the following: automatic gain control (AGC) target value set at 4e5 for the full MS and 5e4 for the MS/MS spectral acquisition, MS1 resolution was set to 120 000 and 30 000 for MS/MS events. Precursor ions were fragmented by stepped high-energy collision dissociation (HCD) using collision energies of 20, 40, and 60. The mass spectrometric data of the analysed betacyanin pigments can be found in Supplementary Table S2.

## Results and discussion

### Selection of BAHD ATs for betanin acylation

ATs of the BAHD acyltransferases (BAHD AT) superfamily have been suggested to be responsible for the acyl-CoA-dependent acylation of betalains in plants, especially for acylation with aliphatic acyl groups (see Fig. 1C). The red pulp of the dragon fruit *H. polyrhizus* contains large amounts of the acylated betacyanins phyllocactin (6′-O-malonyl-betanin) and hylocerenin (6′-O-(3″-hydroxy-3″-methylglutaryl)-betanin) and is therefore expected to express one or multiple BAHD AT involved in betanin acylation. Annotated transcriptome data for betalain-producing plants is limited. Therefore, we started searching for BAHD AT candidate genes by assembling and annotating the publicly available transcriptomes for *H. polyrhizus* in red and white pulp stage. Afterwards, we blasted the protein sequences of two malonyltransferases from soy (GmIF7MAT) and *Arabidopsis thaliana* (At5MAT) into the assembled transcriptomes (D'Auria et al. 2007, Suzuki et al. 2007). Additionally, as BAHD AT-annotated sequences were extracted from the plant transcriptomes. Sequences expressed at least 2-fold in the red pulp stage compared to the white stage were included in the hit list. From those sequences, six were selected based on their high absolute and relative expression levels and named HpBAHD1-HpBAHD6 (Table 1). We also mined the transcriptome of *C. argentea* var. *cristata* (formerly known as *Celosia cristata*) for BAHD AT genes and selected the three highest-expressed candidates: CcBAHD1- CcBAHD3. The final hit list comprised 9 BAHD ATs, ready to be screened in *S. cerevisiae*.

### *In vivo* screening of plant BAHD ATs in *S. cerevisiae*

**Figure 2.**
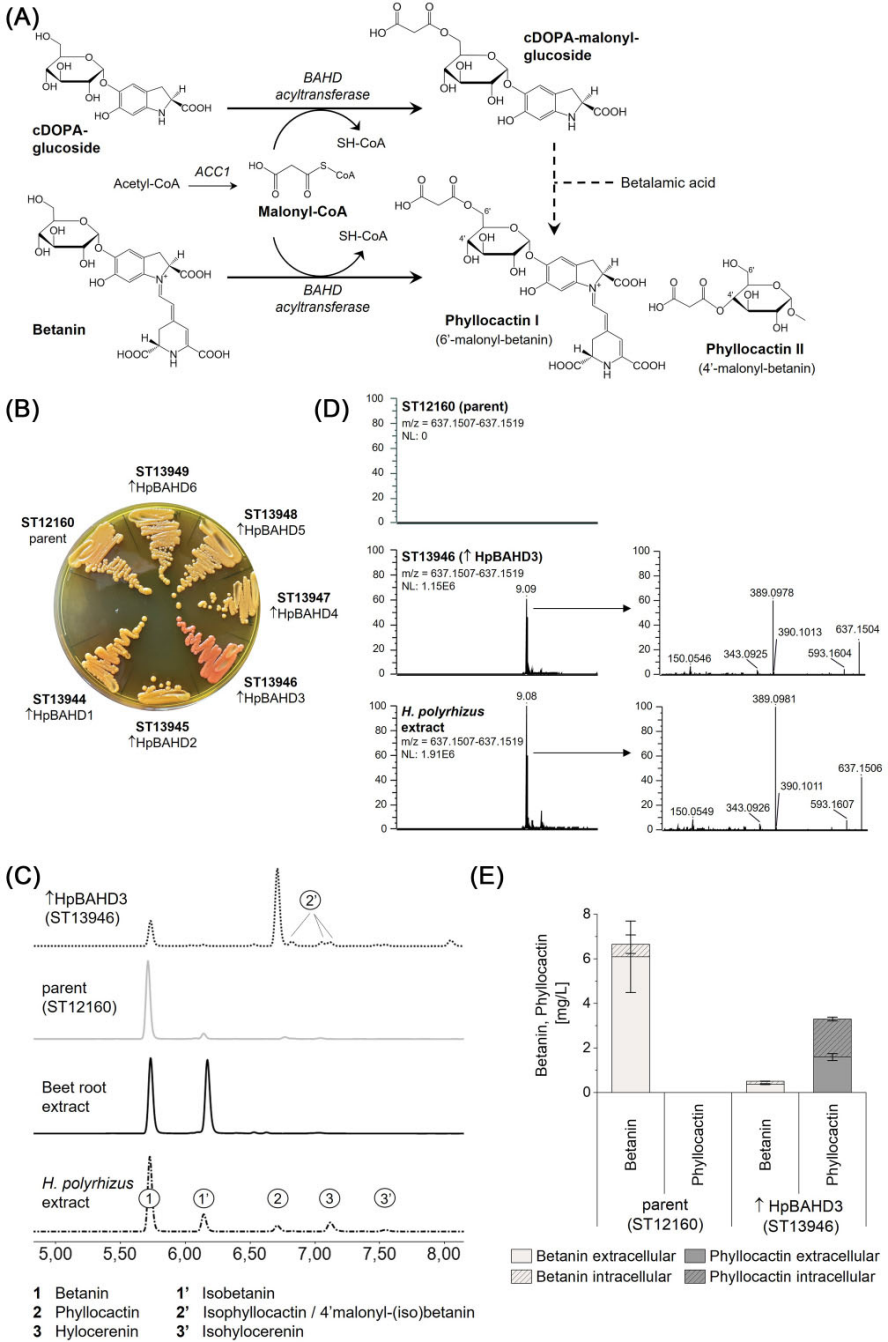
Screening of nine plant BAHD ATs for production of acylated betacyanins in *S. cerevisiae*. (A) Heterologous biosynthesis of phyllocactin in betanin-producing yeast. The integrated BAHD AT catalyses the attachment of malonyl to the 4′- or 6′-hydroxyl group of betanin or cDOPA-glucoside. Malonyl-CoA, produced in the yeast as part of the fatty acid pathway, serves as acyl donor. The 6′-O-position (phyllocactin I) is favoured. ACC = acetyl-CoA carboxylase. (B) *S. cerevisiae* strains on a YPD agar plate, each expressing a different BAHD AT from *H. polyrhizus*. (C) HPLC chromatogram, comparing the supernatant of ST13946 (↑HpBAHD3) and the parent strain ST12160 with a commercial betanin standard from beetroot and *H. polyrhizus* extract at 540 nm. The peak at 6.7 min, present in ST13946 but not in the parent strain ST12160, corresponds to phyllocactin. Since the y-axes have different scales, the signal intensity amongst the samples cannot be compared. (D) LC–MS and MS^2^ analysis of the yeast strains and the dragon fruit extract confirm the presence of phyllocactin (RT 9.09, m/z = 637.1517) in the strain expressing HpBAHD3. (E) Intra- and extracellular betanin and phyllocactin titres produced in small-scale cultivation by ST12160 and ST13946 (↑HpBAHD3). The betanin and isobetanin titres were combined. Mean values of biological triplicates (± SD) are shown.

To screen for production of acylated betalains, we expressed the BAHD AT candidate genes in the betanin-producing *S. cerevisiae* strain ST12160. In a previous work, we engineered a *S. cerevisiae* strain that produced ca. 7 mg/l betanin, expressing one copy of each enzyme involved in betanin biosynthesis: BvTYH^W13L^, MjDOD, and BvGT2 (Babaei et al. 2023). As mentioned, BAHD ATs require a CoA-activated acyl donor. This implies that malonyl-CoA and HMG-CoA must be available in the cell to synthesize phyllocactin (malonyl-betanin) or hylocerenin (HMG-betanin). Like most yeasts, *S. cerevisiae* produces malonyl-CoA as an intermediate of the fatty acid synthesis pathway and HMG-CoA as part of the mevalonate pathway (Basson et al. 1986, Cao et al. 2016, Johnson et al. 2017). We decided that the intracellular levels of both acyl-CoAs are likely not limiting and that additional engineering to increase the concentration of the acyl-donors was not necessary for initial screening. It has not been elucidated yet whether the ATs attach the acyl group to betanin or to cDOPA-glucoside, which then condenses with betalamic acid to form the acylated betacyanin. The suggested biosynthesis pathway for malonylation of betanin in *S. cerevisiae* is illustrated in Fig. 2A.

Integration of the 9 BAHD AT candidate genes into betanin-producing strain ST12160 created strains ST13941-ST13949. After incubation on agar plates, the strain expressing HpBAHD3 (ST13946) was clearly distinct from the other strains by its red colour, while the other strains were of the same light orange colour as the parent strain (Fig. 2B). The yeast strains were cultivated in 2 ml MM for 48 h, and the supernatant and total extract (intra- and extracellular fractions) were analysed by HPLC and compared to plant extract from the red dragon fruit. The HPLC results showed that the main product of the strain expressing HpBAHD3 was a compound with a retention time (RT) of 6.7 min and a λ_max_ of 537 nm, that was also present in the dragon fruit extract (Fig. 2C, peak 2). Further characterization of this compound by mass spectrophotometry showed a peak of 637.1514 m/z [(M + H)^+^] and an MS^2^ fractionation that fit phyllocactin (Fig. 2D). From the LC–MS data, we could not say with certainty which phyllocactin the yeast strains were producing, as all four isoforms (phyllocactin I: 6′-O-malonyl-betanin; isophyllocactin I: 6′-O-malonyl-isobetanin; phyllocactin II: 4′-O-malonyl-betanin; isophyllocactin II: 4′-O-malonyl-isobetanin) have the same MS and MS^2^ profile and only differ in their RT. Based on previous studies that identified 6′-O-malonyl-betanin as the most abundant compound of those four in *H. polyrhizus*, we assumed that the most prominent LC–MS peak with an m/z of 637.1514 and an RT of 9.08 was phyllocactin I (Wybraniec et al. 2007). This peak corresponded to the peak at 6.7 min in the HPLC, indicating that the new compound the yeast produced was phyllocactin I (6′-O-malonyl-betanin). Both yeast strain and dragon fruit extract contained small amounts of the phyllocactin isoforms, as indicated by the small peaks with higher RT than the main peak in the LC–MS chromatogram (Fig. 2D).

It should be mentioned here that acyl migration of the malonyl from C6′ to C4′ and *vice versa* has been shown to be possible in phyllocactin, whereby the 6′-O-position was favoured (Wybraniec 2008). Furthermore, all four isoforms are expected to exhibit identical chromatic properties. Therefore, all isoforms will, from now on, be referred to as phyllocactin unless a differentiation is relevant. The other two peaks in the dragon fruit extract with an RT of 7.12 (3) and 7.54 (3′) in the HPLC (Fig. 2C), had a m/z of 695.1918, corresponding to hylocerenin and isohylocerenin (Supplementary Fig. S1). Neither of the two compounds was detected in one of the yeast strains that expressed one of the nine BAHD ATs. An overview of the chromatographic, spectroscopic, and mass spectrometric data of compounds analysed in this study can be found in Supplementary Table S2.

While the parent strain ST12160 exclusively produced betanin (5.5 mg/l intracellular, 0.7 mg/l extracellular), strain ST13946, expressing HpBAHD3, produced around 3.5 mg/l phyllocactin (1.95 mg/l intracellular, 1.6 mg/l extracellular) and small amounts of betanin (0.5 mg/l) (Fig. 2E). Quantification of phyllocactin was achieved by applying the Beer–Lambert equation to a fraction of phyllocactin purified by semi-preparative HPLC from the *H. polyrhizus* extract. Unlike betanin, which is almost entirely secreted into the cultivation media, 45% of the phyllocactin was retained within the cells. So far, we have not identified the transporter(s) involved in the export of betanin and other betalain variants.

HpBAHD3 was the only one of the nine screened BAHD ATs that produced an acylated betalain variant. No BAHD AT candidate from *C. argentea* was active, even though the plant has been shown to produce phyllocactin in small amounts (Lystvan et al. 2018). Whether the BAHD ATs are not expressed or active in the heterologous system or whether the selected enzymes catalyse other reactions in the plants and are not involved in betalain biosynthesis remained unclear. Also, none of the strains produced hylocerenin. While it is possible that low HMG-CoA concentration in the cells or low expression or activity of the enzyme prevented hylocerenin production, it could also be that none of the selected ATs is involved in hylocerenin synthesis. Returning to the transcriptomic data, we saw that HpBAHD3 was annotated as ‘uncharacterised acetyltransferase At3g50280’. The gene was higher expressed in red than in white pulp stage, and absolute expression was high—but not the highest—compared to other sequences. We assume that HpBAHD3 is involved in betalain biosynthesis in *H. polyrhizus* and alone or with other BAHD ATs responsible for producing phyllocactin in the dragon fruit.

### Expression of HpBAHD3 in betanin-producing *Yarrowia lipolytica*

After we had seen that HpBAHD3 enabled the formation of phyllocactin in *S. cerevisiae*, we set out to investigate whether we could produce the acylated betalain in *Yarrowia lipolytica*. We have recently developed a *Yarrowia* strain (ST12603) that produced high betanin titres (Thomsen et al. 2023). This strain was engineered towards betanin production by integration of three copies of the biosynthetic pathway (MjDOD-EvTYH-BvGT2), feedback-inhibition-resistant Aro4 (YlARO4^K221L^) and Aro7 (YlARO7^G139S^), and deletion of the 4-hydroxyphenylpyruvate acid dioxygenase (Δ4HPPD) that is involved in by-product formation. To enable the production of phyllocactin, we integrated one copy of the codon-optimized HpBAHD3 into the platform strain ST12603, resulting in strain ST14103.

**Figure 3.**
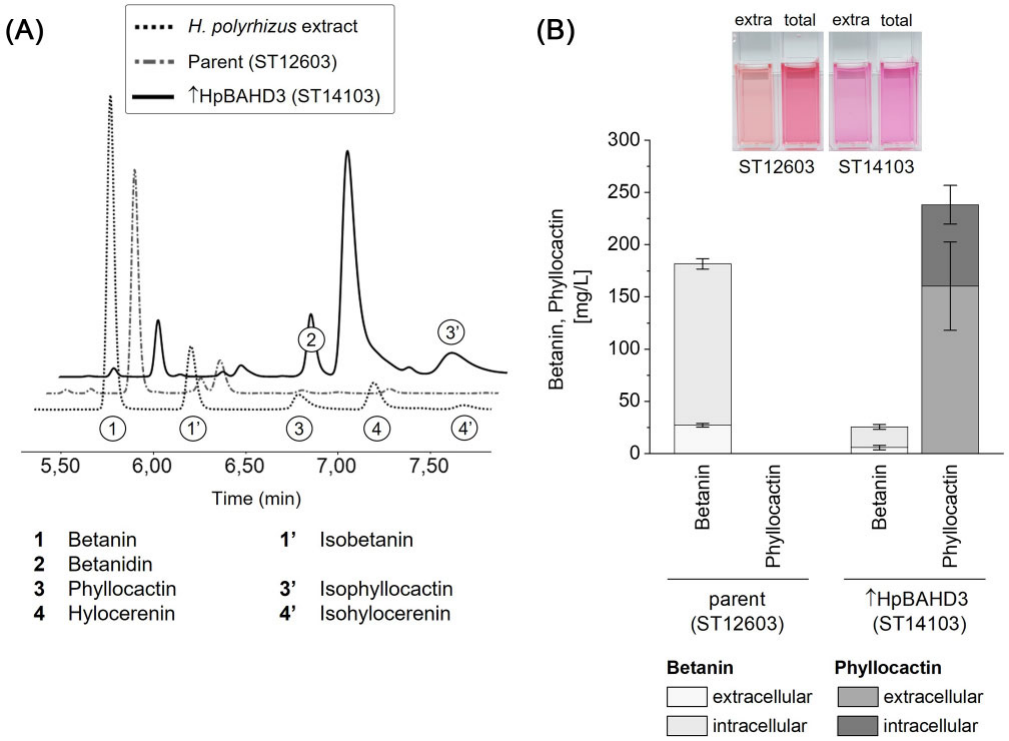
Phyllocactin production with *Y. lipolytica*. ST12603 (parent) and ST14103 (+ HpBAHD3) were cultivated in MM in 24-well plates for 48 h. (A) 1:10 diluted total fractions were analysed by HPLC and compared to dragon fruit extract. The betalain variants were identified with standards made from plant extracts and confirmed by LC–MS. (B) Intra- and extracellular concentrations of betanin and phyllocactin. The concentrations of C15 stereoisomers were combined. The 1:10 diluted extracellular and total fractions illustrate the difference in hue between the betanin- and the phyllocactin-producing strain and the increased titre of betanin after lysis of the cells in the total fraction of ST12603. Mean values of biological triplicates (± SD) are shown.

The strains were assessed in small-scale cultivation in 2 ml MM in 24-well plates. HPLC (Fig. 3A) and LC–MS analysis (Supplementary Fig. S2) confirmed that also in *Yarrowia*, HpBAHD3 enabled the formation of phyllocactin. As expected, the heavily engineered parent strain (ST12603) produced considerably higher titres of betanin than the betanin-producing *S. cerevisiae* strain (ST12160). We were surprised, however, to see that not only most of the betanin was converted to phyllocactin but that ca. 250 mg/l (0.389 mM) phyllocactin was produced (Fig. 3B). The parent strain, in comparison, produced only 180 mg/l (0.33 mM) of betanin. There was a big difference between the intra- and extracellular concentrations of betanin and phyllocactin. While the majority of betanin remained in the yeast cells and only 15% (ST12603) to 25% (ST14103) of betanin were secreted into the media, ca. 70% of the phyllocactin was detected in the extracellular fraction. In *S. cerevisiae*, the opposite trend had been observed, and while betanin was almost entirely secreted, half of the phyllocactin was retained in the cells (Fig. 2E). Babaei et al. (2023) compared betanin secretion in two *S. cerevisiae* strains with different genetic backgrounds (CEN.PK113-5D and S288c) and observed a difference in secretion efficiency between the strains. Thus, it is conceivable that different transport mechanisms between *S. cerevisiae* and *Y. lipolytica* might favour the export of betanin or phyllocactin, respectively.

While the dragon fruit contains a mix of betanin, hylocerenin, and phyllocactin, the strain expressing HpBAHD3 produced almost exclusively phyllocactin and only small amounts of betanin and betanidin. No hylocerenin could be detected in the *Yarrowia* strain (Supplementary Fig. S3). The results of the small-scale cultivation demonstrated a high efficiency of HpBAHD3 to acylate betanin, allowing a higher titre of phyllocactin than of betanin.

The remaining eight BAHD ATs were not tested in *Y. lipolytica*, as our primary focus at this stage was enhancing phyllocactin production. However, the different genetic background and metabolite pools of the oleaginous yeast could influence the screening and enable the formation of another acylated betanin derivative. For example, the high HMG-CoA concentrations in *Y. lipolytica* might promote hylocerenin production by one of the other ATs, which could not be detected in *S. cerevisiae*. Consequently, screening these enzymes in *Y. lipolytica* should be considered.

### Fed-batch fermentation enables the gram-scale phyllocactin production

To assess the potential of phyllocactin production on a larger scale, fed-batch fermentations of the phyllocactin-producing strain (ST14103) and its parent strain (ST12603) were performed. The fermentations were carried out in duplicates in 250 ml AMBR bioreactors in minimal medium at pH 6 with glucose as carbon source. As whole-cell samples were directly frozen after sampling, only the total betacyanin production could accurately be determined. The raw data for the fermentations can be found in the Supplementary File S3, the online process data in Supplementary Fig. S4.

**Figure 4.**
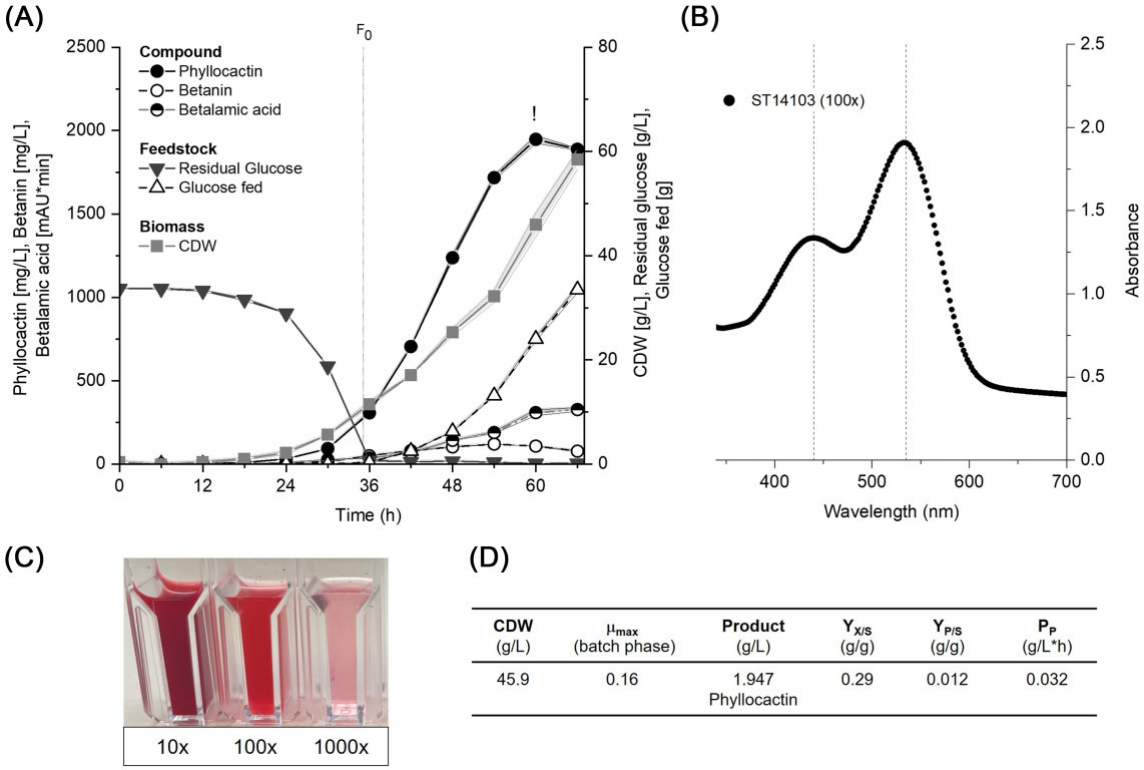
Fed-batch fermentation of the phyllocactin-producing *Y. lipolytica* strain ST14103 in 250 ml bioreactors in duplicates. (A) Betacyanin titres, biomass production, and feedstock development over 66 h of fermentation. Solid lines indicate the average from both bioreactors, shaded areas represent the corresponding standard deviations. The sample with the highest phyllocactin titre (1.95 g/l) is marked (!). F_0_ = feed initiation. (B) UV-vis spectrum of the 100× diluted sample after 66 h. (C) 10×, 100×, and 1000× diluted samples after 66 h. (D) Relevant process data after 60 h (highest titre) of fermentation.

Unfortunately, both replicates of ST12603 were aborted after 35 h and 40 h of cultivation due to excessive foaming and the delayed automatic addition of antifoam by the AMBR250 system (Supplementary Fig. S5). The fermentation of ST14103 ran for 66 h, whereby the highest phyllocactin titre was reached after 60 h, at which point the yeast had produced 1.947 g/l ± 24 mg/l phyllocactin (Fig. 4A). HPLC analysis and UV-vis spectrum of the broth showed that in addition to the red betacyanins phyllocactin and betanin (λ_max_ = 535 nm), the strain produced betalamic acid (λ_max_ = 410 nm) and betaxanthins (λ_max_ ≈ 480 nm) (Fig. 4B, Supplementary Fig. S6). In ST12603, the UV-vis spectrum showed a higher absorbance at 450 nm than at 535 nm, visible as an orange-red colour of the broth (Supplementary Fig. S5). In the phyllocactin-producing strain, in contrast, the ratio between the two peaks favoured the betacyanin peak, resulting in a red-pink colour of the broth, even when highly diluted (Fig. 4C).

Thomsen et al. (2023) reported a betanin titre of 1.2 g/l in an almost identical fermentation process and with a strain with the same modifications as ST12603. There, betanin titres were highest after 48 h of fermentation and decreased afterwards. Upon disruption of two beta-glucosidases, the titre could be increased to 1.3 g/l in 51 h. The phyllocactin titres exceed these betanin titres by >50%, even though no beta-glucosidases were disrupted. This is remarkable, considering that the formation of one molecule of phyllocactin requires one molecule of betanin and an additional molecule of malonyl-CoA.

There are several explanations why more phyllocactin was produced than betanin. As an oleaginous yeast, *Y. lipolytica* is naturally endowed with high fluxes towards malonyl-CoA and the pentose phosphate pathway (PPP), metabolic traits relevant for the production of natural products requiring aromatic amino acids and malonyl-CoA building blocks (Blank et al. 2005, Wasylenko et al. 2015, Sáez-Sáez et al. 2020). However, this alone cannot explain the higher production of phyllocactin compared to betanin. Another explanation is that an enhanced stability of the acylated compound contributes to the observed titres. Contradicting previous assumptions, Herbach et al. (2006a) showed that purified phyllocactin is slightly less stable than betanin towards heat-induced degradation. However, degradation is influenced by multiple other parameters such as pH, exposure to O_2_ and light, water activity (a_w_), and the presence of metal cations and degrading enzymes such as oxidases, beta-glucosidases, or peroxidases (Herbach et al. 2006b). Purified phyllocactin has shown a higher tendency towards degradation by de-acylation and decarboxylation than hydrolysis of the aldimine bond, resulting in betanin and decarboxylated derivatives (Herbach et al. 2006a). Since betanin is more stable than betalamic acid and cDOPA-glucoside derivatives, it might be re-acylated by the BAHD AT to form phyllocactin, while degradation of betanin results in the formation of betaxanthins and unstable degradation products. Alternatively, the acylated cDOPA-glucoside could be more stable than the unacylated cDOPA derivative and, therefore, be more likely to recondense with betalamic acid to form phyllocactin. Both hypotheses would also explain the difference in the UV-vis spectra of the *Yarrowia* strains. Alternatively, the acylated betacyanin could be more resistant to enzymatic degradation. As described for malonylated anthocyanins, the acyl group might protect phyllocactin from endogenous beta-glucosidases or oxidases of the yeast and thereby prevent its degradation during cultivation (Suzuki et al. 2002).

Further characterization of the microbially produced phyllocactin is needed to assess its stability and colour properties under varying conditions, particularly given the limited data available in the literature. However, so far, we have not observed a significant difference in stability between purified fractions of betanin and phyllocactin. Therefore, the primary advantages of phyllocactin are expected to lie in (i) its superior stability during the fermentative production, leading to higher titres compared to betanin, and (ii) its enhanced pigment integrity, likely attributed to the partially red degradation products (Herbach et al. 2006a).

During the fed-batch fermentation, phyllocactin made up 95% of the produced betacyanin (1947 mg/l; 3.1 mM), while only 5% accounted for betanin (107 mg/l; 0.2 mM) (Fig. 4, Supplementary Fig. S6). The dragon fruit extract, in contrast, contains large amounts of betanin and hylocerenin in addition to phyllocactin (Fig. 3A), whereby the exact composition and distribution vary between different *Hylocereus* species (Esquivel et al. 2007). This high purity is an advantage of the heterologous phyllocactin production in yeast over its extraction from dragon fruit. High purity is a desirable trait for natural colours in the food industry, as it facilitates precise determination of the dye’s properties, ensures consistent colouring, and simplifies dosage requirements. Beetroots, for example, mainly contain betanin, isobetanin, and vulgaxanthin and only negligible amounts of other betaxanthins and betacyanins, which makes them a convenient and consistent source of betalains (Nagy-Gasztonyi et al. 2001, Slavov et al. 2013, Guldiken et al. 2016). Other betalain-producing plants such as dragon fruit (*Hylocereus* spp.), amaranth *(Amaranthus spp.)*, quinoa *(Chenopodium quinoa), Gomphrena globosa*, or *Bougainvillea* spp. produce a mixture of different betalains, complicating their use as food dyes (Piattelli and Imperato 1970a, 1970b, Cai et al. 2001, Wybraniec et al. 2001, Stintzing et al. 2002; Kugler et al. 2007, Escribano et al. 2017, Lystvan et al. 2018).

Recombinant betanin production has been reported in *E. coli, S. cerevisiae, Y. lipolytica*, and *Fusarium venenatum* as well as in several recombinant plants. A few betalain variants have been produced before, but exclusively in plant cells. Imamura et al. (2019) reported the successful production of 14 µM (10.2 mg/l) amaranthin in tobacco BY-2 cells and of celosianin II in quinoa and amaranth. Polturak et al. (2018) detected aromatically acylated betanin variants in *N. benthamiana* leaves after integrating an HCGT from *M. jalapa* and the biosynthetic pathway for betanin into the plant. Interestingly, production of these acylated betalains was possible without overexpression of a betalain-related AT, suggesting promiscuous betanin-AT activity of unknown HCG-dependent *N. benthamiana* ATs. Heterologous phyllocactin production, however, has never been reported before in either plant or microbial host. Beyond being non-toxic food colourants, increasing research highlights the numerous health benefits of betalains. Their antioxidant and antiradical properties, which protect against oxidative damage, have been recognized for a long time and even surpass the free radical-scavenging activity of anthocyanins and vitamin C (Escribano et al. 1998, Kanner et al. 2001, Gengatharan et al. 2015). Additionally, studies have demonstrated that betalains possess antiproliferative, antiviral, antimicrobial, and anti-lipidemic effects (Sani et al. 2009, Tenore et al. 2012, Esatbeyoglu et al. 2014, Lim et al. 2024). For instance, daily consumption of a drink of red dragon fruit powder for 14 days, containing 33 mg of betalains, improved cardiovascular function in both men and women by enhancing endothelial function and reducing arterial stiffness (Cheok et al. 2022). In another study, *H. polyrhizus* extract was shown to inhibit the growth of melanoma cancer cells in a dose-dependent manner in cell cultures (Wu et al. 2006). These findings demonstrate the appeal and necessity of making betalains more widely available, a process that biotechnological production could facilitate.

## Conclusion

In this study, we explored the transcriptomes of red-fleshed dragon fruit (*H. polyrhizus*) and cockscomb (*C. argentea* var. *cristata*) to identify BAHD acyltransferases potentially involved in betanin acylation. By screening the selected enzymes in betanin-producing *S. cerevisiae*, we discovered that acyltransferase HpBAHD3 from *H. polyrhizus* catalysed the production of phyllocactin. Expression of this enzyme in a *Y. lipolytica* strain engineered for betanin production resulted in the production of high phyllocactin titres with minimal betanin formation. In lab-scale fed-batch fermentation, 2 g/l of phyllocactin was obtained, making fermentation an attractive process for phyllocactin production. Considering that 100 g of fresh dragon fruit pulp contains 25–42 mg betacyanins, of which ca. one-third is phyllocactin, disregarding potential product loss during extraction or downstream processing, and assuming a weight of 400 g per peeled fruit, 36–60 fruits or 14–24 kg would be required to obtain those 2 g of phyllocactin (Stintzing et al. 2002, Esquivel et al. 2007, Nurul and Asmah 2014, Rodriguez et al. 2016).

## Supplementary Material

foae041_Supplemental_Files
