# Supplementary material for: BAHD acyltransferase from dragon fruit enables production of phyllocactin in engineered yeast
Source: FEMS Yeast Res. 2025 Feb 10;25:foae041. doi: 10.1093/femsyr/foae041 (PMC11881927; doi:10.1093/femsyr/foae041)
Supplement: foae041_Supplemental_Files [file foae041_supplemental_files.zip › Supplementary File 1_revision_clean.docx]

**Supplementary File 1**

[**Table S1.** Raw data for the calculation of calibration curves for the described betacyanin variants. 2](#_Toc178768639)

[**Table S2**. Chromatographic, spectroscopic and mass spectrometric data of compounds detected and quantified in this manuscript. 2](#_Toc178768640)

**Figure S1**. LC-MS and MS^2^ data of dragon fruit extract *(H. polyrhizus*) and *S. cerevisiae* strain ST13946, expressing the acyltransferase HpBAHD3, for hylocerenin. . 3

**Figure S2**. LC-MS and MS^2^ data confirming phyllocactin production by Y. *lipolytica* strain ST14103 expressing HpBAHD3. 3

**Figure S3.** LC-MS and MS^2^ data of *Y. lipolytica* ST14103 and of *H. polyrhizus* fruit extract for hylocerenin. 4

**Figure S4**. Process data for fed-batch fermentation of ST14103 in 250mL AMBR bioreactor. 4

**Figure S5**. Fed-batch fermentation of the betanin-producing Yarrowia strain ST12603 in 250 mL bioreactors. 5

**Figure S6**. HPLC chromatogram of ST14103 (↑HpBAHD3) after 60 h of fermentation in the bioreactor (250 mL). 6

**Table S1.** Raw data for the calculation of calibration curves for the described betacyanin variants. From the absorbance values of pure compounds (commercial standards or purified from plant extracts) and their peak area (mAU*min) in the HPLC, the concentration of the samples was determined and used to calculate a calibration curve for each compound.

| **Compound** | **e (L/cm*mol)** | **MW (g/mol)** | **mg/L** | **μmol/L** | **mAU*min** | **Slope m (b = 0)** |
| --- | --- | --- | --- | --- | --- | --- |
| **Betanin** | 65000 | 550 |  |  |  | **0.703** |
| Betanin 1 |  |  | 0.41 | 0.74 | 0.27 |  |
| Betanin 2 |  |  | 0.81 | 1.47 | 0.54 |  |
| Betanin 3 |  |  | 1.59 | 2.89 | 1.09 |  |
| Betanin 4 |  |  | 3.91 | 7.11 | 2.63 |  |
| Betanin 5 |  |  | 7.77 | 14.13 | 5.53 |  |
| Betanin 6 |  |  | 15.50 | 28.18 | 10.9 |  |
| **Isobetanin** | 65000 | 550 |  |  |  | **0.719** |
| Isobetanin 1 |  |  | 0.41 | 0.74 | 0.28 |  |
| Isobetanin 2 |  |  | 0.81 | 1.47 | 0.56 |  |
| Isobetanin 3 |  |  | 1.59 | 2.89 | 1.12 |  |
| Isobetanin 4 |  |  | 3.91 | 7.11 | 2.68 |  |
| Isobetanin 5 |  |  | 7.77 | 14.13 | 5.66 |  |
| Isobetanin 6 |  |  | 15.50 | 28.18 | 11.15 |  |
| **Phyllocactin** | 65000 | 637.5 |  |  |  | **0.600** |
| Phyllocactin 1% |  |  | 2.37 | 3.72 | 1.81 |  |
| Phyllocactin 10% |  |  | 23.73 | 37.23 | 13.55 |  |
| Phyllocactin 25% |  |  | 55.90 | 87.69 | 33.05 |  |
| Phyllocactin 50% |  |  | 109.85 | 172.31 | 67.75 |  |
| Phyllocactin 100% |  |  | 237.35 | 372.31 | 141.81 |  |

**Table S2**. Chromatographic, spectroscopic and mass spectrometric data of compounds detected and quantified in this manuscript. Max. absorbance differs between sources.

| **Compound** | **Trivial name** | **Molecular weight [g/mol]** | **λ_max_ (nm)** | **Retention time [min] (HPLC)** | **m/z [M+H]^+^** | **MS^2^ (expected)** |
| --- | --- | --- | --- | --- | --- | --- |
| Betanidin | Betanidin | 388.3 | 545^[1]^ | 6.5 | 389.0984 | 325; 297; 255; 241; 149 |
| Betanidin 5-O-β-glucoside | Betanin | 550.5 | 535^[1,2]^ | 5.7 | 551.1513 | 389; 343; 150 |
| Isobetanidin 5-O-β-glucoside | Isobetanin | 550.5 | 535^[1,2]^ | 6.15 | 551.1513 | 389; 343; 150 |
| Betalamic acid | Betalamic acid | 211.17 | 410^[3]^ | 6.68 |  |  |
| 6’-O-Malonyl-betanin | Phyllocactin (I) | 637.5 | 533^[1]^  537^[2]^  539^[4]^ | 6.7 | 637.1517 | 619; 593; 551; 389; 150 |
| 6’-O-Malonyl-isobetanin | Isophyllocactin (I) | 637.5 | 533^[1]^  537^[2]^  538^[4]^ | 7.12 | 637.1517 | 619; 593; 551; 389; 150 |
| 4’-O-Malonyl-betanin | Phyllocactin II | 637.5 | 533^[1]^ | 6.82 | 637.1528 | 619; 593; 551; 389; 150 |
| 4’-O-Malonyl-isobetanin | Isophyllocactin II | 637.5 | 533^[1]^ | ? | 637.1528 | 619; 593; 551; 389; 150 |
| 6’-O-(3’’-Hydroxy-3’’-methylglutaryl)-betanin | Hylocerenin | 694.6 | 541^[4]^ | 7.12 | 695.1879 | 677; 651; 633; 551; 389 |
| 6’-O-(3’’-Hydroxy-3’’-methylglutaryl)-isobetanin | Isohylocerenin | 694.6 | 540^[4]^ | 7.54 | 695.1879 | 677; 651; 633; 551; 389 |


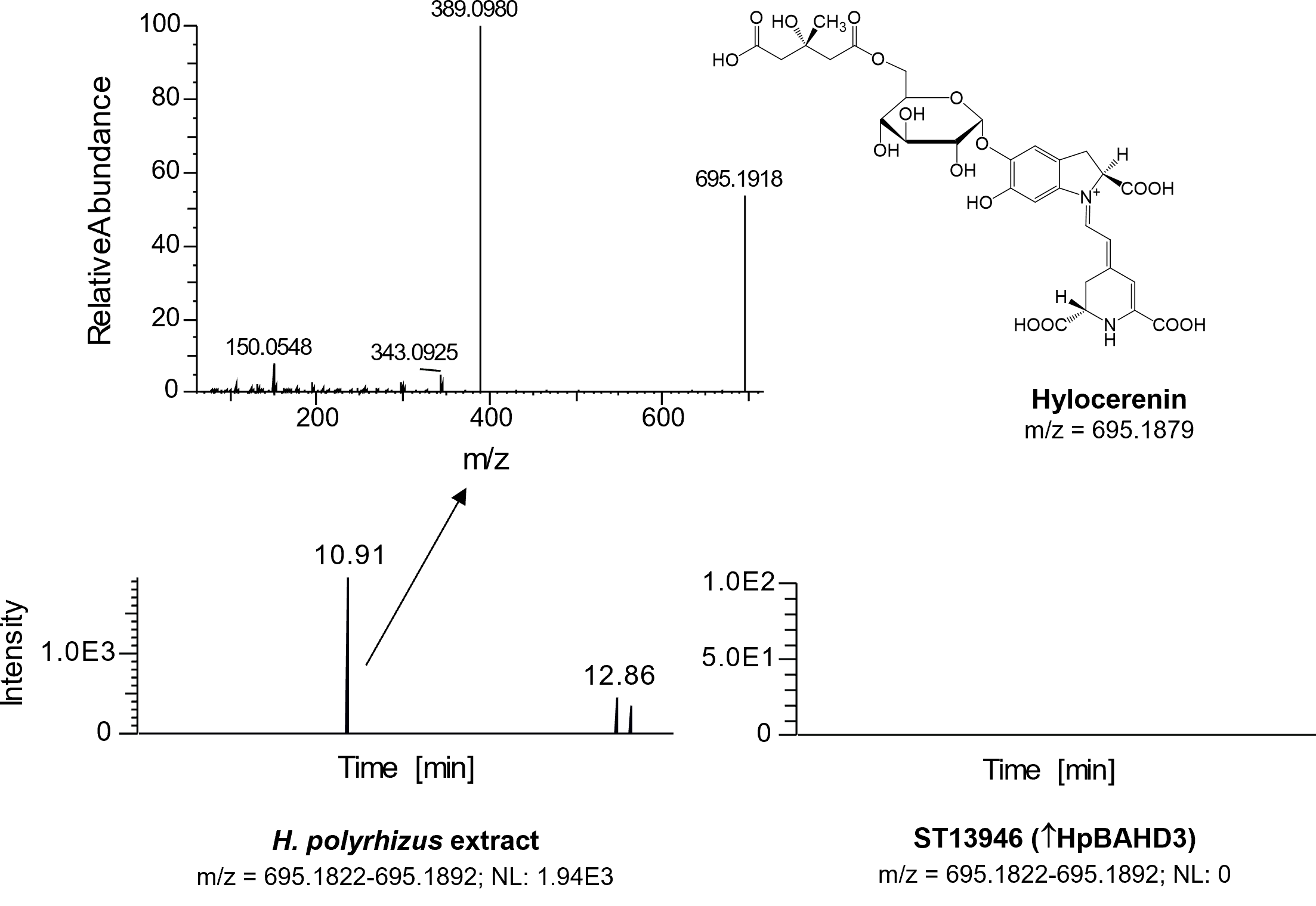


**Figure S1**. LC-MS and MS^2^ data of dragon fruit extract *(H. polyrhizus*) and *S. cerevisiae* strain ST13946, expressing the acyltransferase HpBAHD3, for hylocerenin. In the plant extract, a compound with m/z = 695.1918 was detected, corresponding to hylocerenin. The MS^2^ fragmentation pattern, characteristic for betacyanins, confirmed the identity of hylocerenin (RT 10.91 min). Hylocerenin was not detected in the yeast strain.


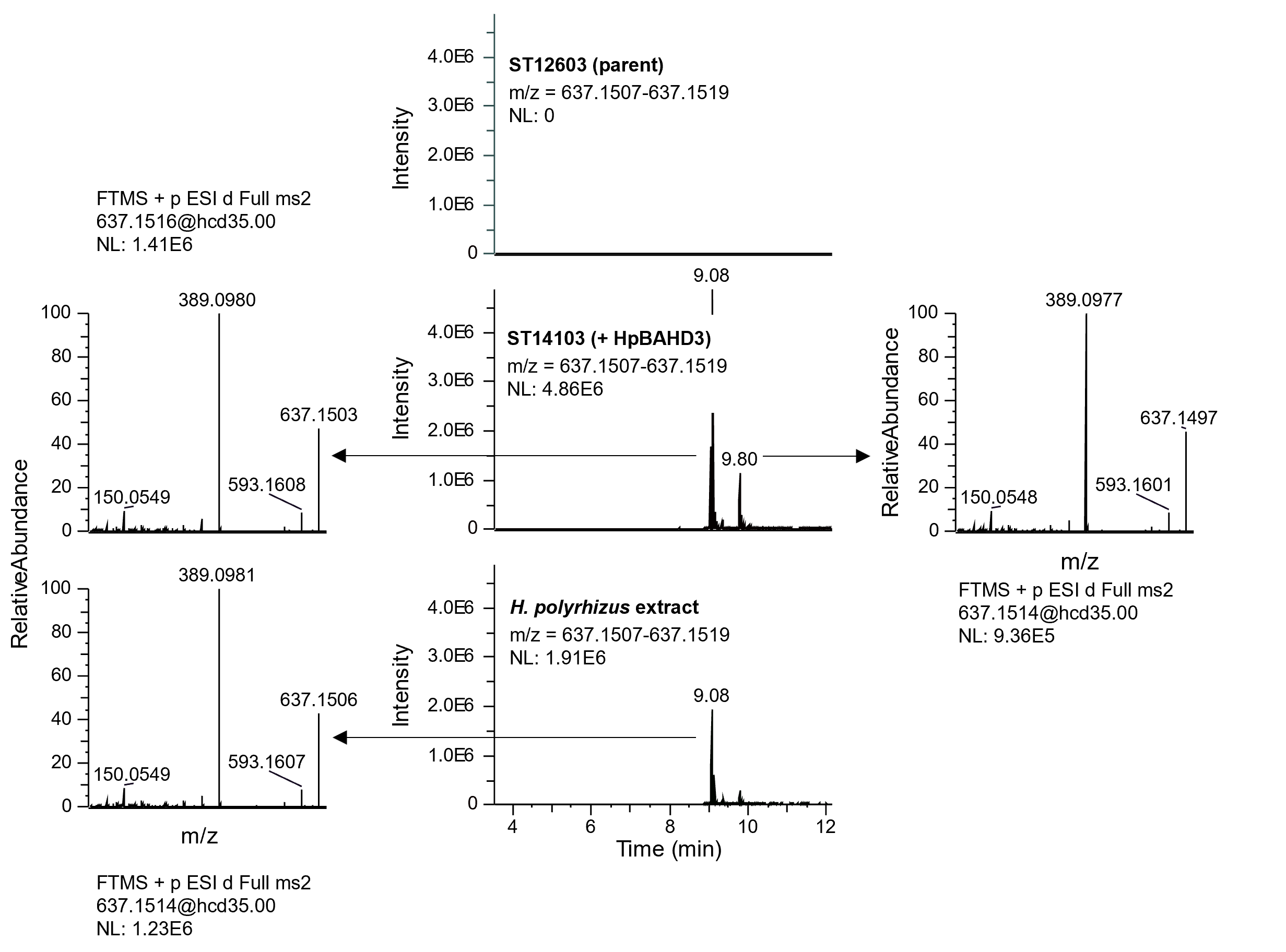


**Figure S2**. LC-MS and MS^2^ data confirming phyllocactin production by Y. *lipolytica* strain ST14103 expressing HpBAHD3. The LC-MS data for the *Y. lipolytica* strains ST12603 (parent) and ST14103 (↑HpBAHD3), cultivated for 48 h in 2 mL MM, and the plant extract from the red dragon fruit *H. polyrhizus* showed that the strain expressing HpBAHD3 produced a compound with m/z = 637.1514, corresponding to phyllocactin. The fragmentation pattern of the MS^2^, characteristic for betacyanins, confirmed the identity of phyllocactin (RT 9.08 min) and one isoform, likely isophyllocactin (RT 9.80 min). Both peaks were also detected in the plant extract. No MS^2^ was available for isophyllocactin in the dragon fruit extract but the RT matched to the RT (9.80 min) of isophyllocactin in ST14103.

**Figure S3.** LC-MS and MS^2^ data of *Y. lipolytica* ST14103 and of *H. polyrhizus* fruit extract for hylocerenin. In the plant, two compounds with m/z = 695.1918 and m/z = 695.1923 were detected, corresponding to hylocerenin (RT 10.9 min) and presumably its C15 stereoisomer isohylocerenin (RT 11.47 min). No hylocerenin was detected in the *Y. lipolytica* strain.

**Figure S4**. Process data for fed-batch fermentation of ST14103 in 250mL AMBR bioreactor. The feed was started upon expected depletion of glucose in the batch medium, indicated by a drop in the CER.


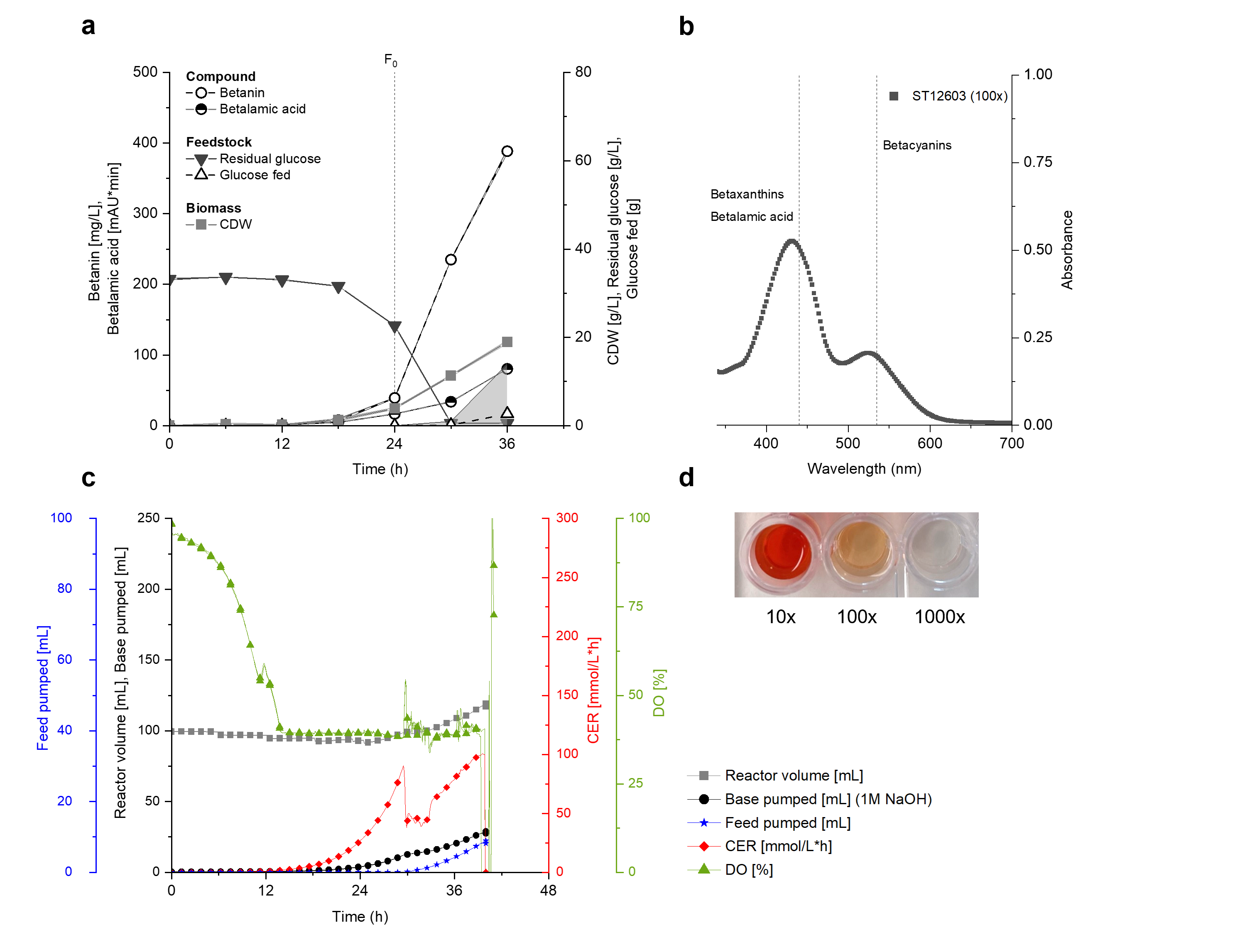


**Figure S5**. Fed-batch fermentation of the betanin-producing Yarrowia strain ST12603 in 250 mL bioreactors. Due to excessive foaming after 35 h and 40 h, both replicates were stopped before the fermentation was finished. **a** Betacyanin titers, biomass production and feedstock development over 36 h of fermentation. Solid lines indicate the average from both bioreactors, shaded areas represent the corresponding standard deviations. **b** UV-vis spectrum of the 100x diluted sample after 36 h. The line at ca. 450 nm corresponds to betalamic acid and betaxanthins, the line at 535 nm to betacyanins **c** Relevant online process data of both replicates **d** 10x, 100x and 1000x diluted sample after 36 h.


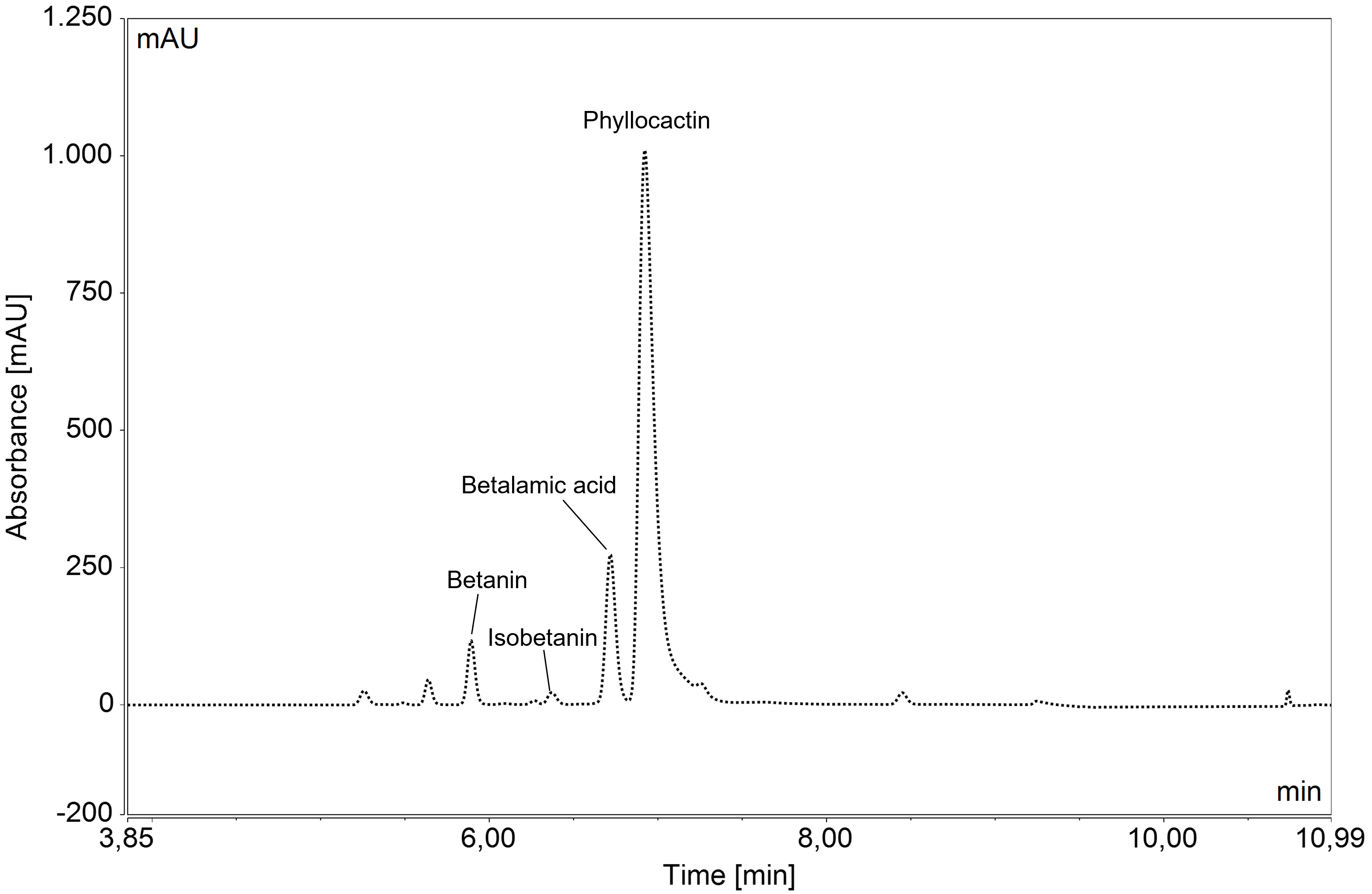


**Figure S6**. HPLC chromatogram of ST14103 (↑HpBAHD3) after 60 h of fermentation in the bioreactor (250 mL). Absorbance at 540 nm of the total production (intra- and extracellular) is shown. Even though the λ_max_ of betalamic acid is at 410 nm, the fringes are detectable at 540 nm.
